# Vergence Formula for Estimating the Refractive Status of Aphakic Eyes in Pediatric Patients

**DOI:** 10.3389/fmed.2022.861745

**Published:** 2022-04-08

**Authors:** Linlu Tian, Peiquan Zhao, Huang Zhu, Xiaoli Kang, Yan Wei, Luya Chen, Jing Li

**Affiliations:** ^1^Department of Ophthalmology, Xinhua Hospital Affiliated to Shanghai Jiao Tong University School of Medicine, Shanghai, China; ^2^Eye Institute and Department of Ophthalmology, Eye & ENT Hospital Affiliated to Fudan University, Shanghai, China; ^3^Department of Ophthalmology, Children Hospital Affiliated to Shanghai Jiao Tong University, Shanghai, China

**Keywords:** aphakia, refraction, vergence formula, pediatric, biometry

## Abstract

**Clinical Relevance:**

A vergence formula may provide a simple and reliable calculation of the refractive status of aphakic eyes.

**Background:**

Measuring the refractive error of pediatric eyes with aphakia is difficult. This study investigated the accuracy and applicability of a vergence formula for estimating the refractive status of such eyes.

**Methods:**

A retrospective review of the medical records, created between January 2016 and December 2018, of pediatric patients with aphakia was conducted. A vergence formula, based on axial length, was used to calculate the refractive status of the aphakic eyes. The refractive values determined using retinoscopy, an automatic refractometer, and the vergence formula were compared.

**Results:**

A total of 72 eyes (47 patients) were analyzed. The spherical equivalents of the refractive errors (mean ± standard deviation) of the eyes were determined using retinoscopy (13.01 ± 3.27 D), automatic refractometry (12.90 ± 3.23 D), and the vergence formula (12.70 ± 3.4 D). The correlation coefficient between retinoscopy values determined using retinoscopy and the vergence formula, automatic refractometry and the vergence formula, and retinoscopy and automatic refractometry were 0.968, 0.987, and 0.979, respectively. The Bland-Altman consistency analysis revealed that the mean differences in the spherical equivalent values between retinoscopy and automatic refractometry, retinoscopy and the vergence formula, and automatic refractometry and the vergence formula were 0.11 D, 0.31 D, and 0.21 D, respectively, with 95% limits of agreement of−1.20 to 1.41 D,−1.37 to 2.00 D, and−0.90 to 1.31 D, respectively.

**Conclusion:**

The vergence formula was effective for evaluating the refractive status of aphakic eyes in pediatric patients.

## Introduction

Surgical treatment of congenital ocular disease is best performed early (1). However, many difficulties arise when implanting intraocular lenses in infants with congenital cataracts, including the difficulty of the operation, serious postoperative trauma, the increased possibility of visual axis opacification, posterior capsular opacification, and the choice of intraocular lens power. Many surgeons prefer using primary posterior capsulectomy and anterior vitrectomy, without intraocular lens implantation, for infants with congenital cataracts (2–5). However, if a primary intraocular lens is not implanted, the resulting amblyopia needs to be treated as soon as possible to facilitate visual rehabilitation. This can be achieved with aphakic glasses or contact lenses (silicone elastomeric lenses or rigid gas permeable lenses) (6–8).

Pediatric aphakia is mainly caused by congenital conditions, such as congenital cataracts, and defects in ocular development. Injuries are less common causes. While the timely implantation of an artificial lens is important, the circumstances sometimes necessitate a prolonged aphakic period. In such cases, accurate assessment of the refractive status becomes critical for vision rehabilitation. Currently, retinoscopy and automatic refractometry are the main assessment approaches. For infants and very young children, who are unable to communicate effectively with medical professionals, retinoscopy is the only way to determine the refractive status of the eye, using chloral hydrate when necessary. However, in patients with visual axis deviations, large refractive errors, corneal or vitreous opacity, irregular corneal surfaces or pupillary shapes, albinism, nystagmus, and retinal conditions such as retinal folds or retinal detachment, retinoscopy assessments become more difficult and the results may not be reliable (9, 10).

**Figure 1:**
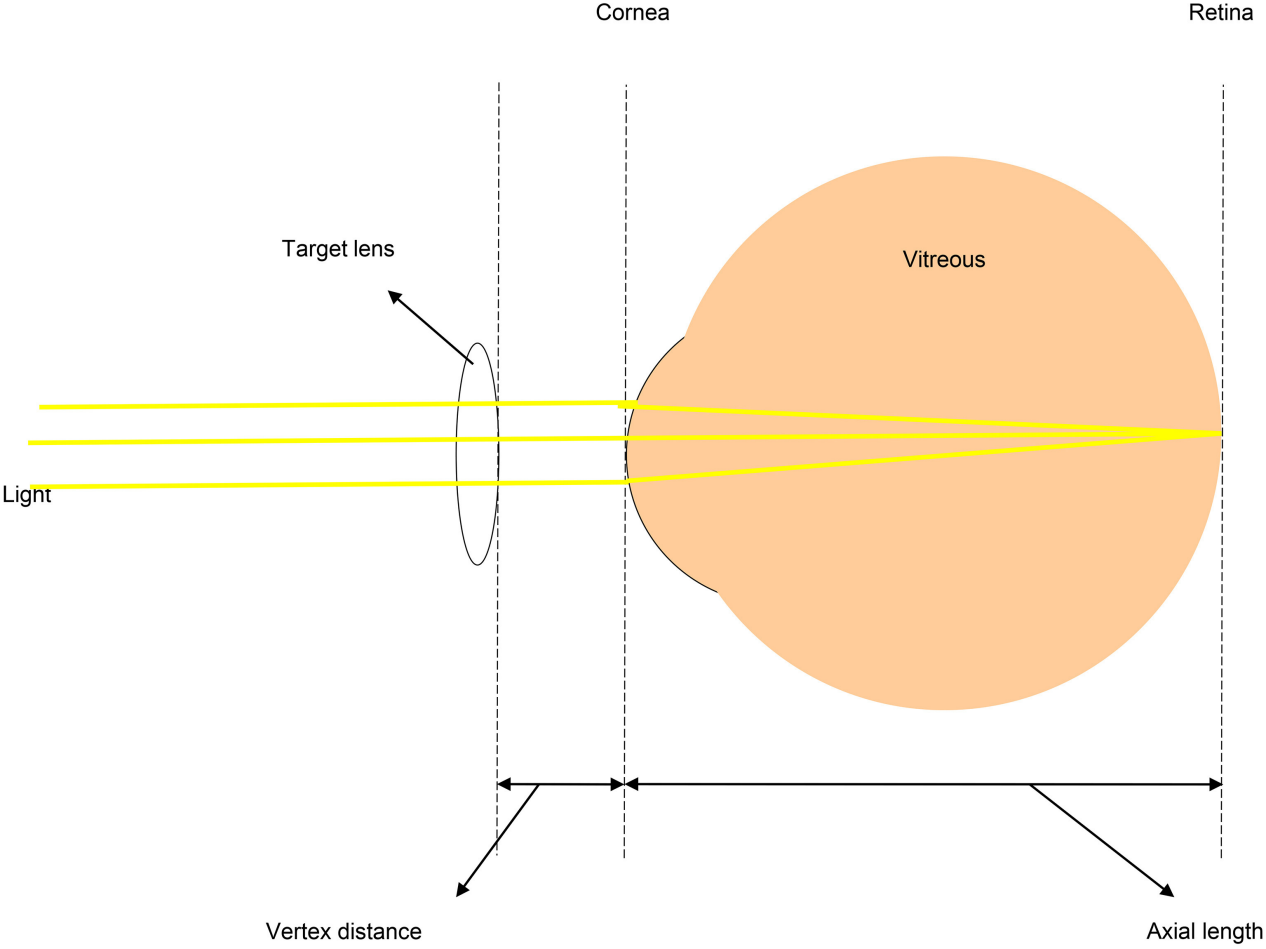
Schematic diagram showing paraxial rays focused on the retina of an aphakic eye. In aphakic eyes, the main refractive media are the cornea and vitreous cavity. If these two structures are regarded as a combined pair of thin lenses, the refractive value of the eyeball can be calculated using a simplified vergence formula.

In aphakic eyes, the cornea and vitreous cavity are the primary structures involved in refraction. Considering both structures as thin lenses, the refractive status of an aphakic eye can be calculated using a simplified vergence formula. If a thin lens is added to the optical surface of the cornea, then parallel light entering the eye can be focused on the retina. The thin lens is then converted to the diopteric power of the target lens based on the vertex distance. Figure 1 is a schematic presentation of the focusing of paraxial rays on the retina using a combination of lenses.

The actual refractive power of the cornea can be calculated using the thick-lens formula (11): $F=\;f_{1}+f_{2}-f_{1}\times f_{2}\times \frac{n}{t}$. However, in children, accurately determining corneal thickness and the curvature of the posterior corneal surface is difficult and time-consuming. An alternative approach is to use the keratometry value, which is calculated considering the cornea as a single refractive surface (11). The keratometry value estimates the total corneal power, based on the anterior corneal curvature and the keratometric index of refraction. The common index of refraction is 1.3375, representing the power at the posterior vertex of the cornea. Determining whether the use of corneal biometry is more accurate than using keratometry for calculating the true refractive power of the cornea is difficult. However, the latter is more manageable and efficient since obtaining the anterior corneal curvature, using a keratometer or a handheld device, is relatively easy.

According to the sign rule, using the cornea as the datum plane allows the restatement of the formula: $F_{C}=\frac{n_{a}}{L}-K$; $F_{e}=\frac{F_{C}}{1+dF_{C}}$

Where:

*F*_*C*_ = estimated refractive value (spherical equivalent value) of the cornea (D, dioptres).

*F*_*e*_ = estimated refractive value (spherical equivalent value) of aphakic glasses (D, dioptres).

d = vertex distance (0.012 m for Asians).

L = axial length (m).

K = average keratometry value (D, dioptres).

*n*_*a*_ (aqueous index of refraction) = 1.336.

Thus, this leads to a final formula: $F_{e}=\frac{1.336/L-K}{1+0.012\left(1.336/L-K\right)}$

In the present study, this formula was used to calculate the refractive status of aphakic children. This allowed a retrospective comparison of results obtained using conventional methods (retinoscopy and automatic refractometry) and the vergence formula.

## Methods

This retrospective study was reviewed and approved by the ethics committee of Xinhua Hospital, affiliated with the Shanghai Jiao Tong University School of Medicine (Approval No. XHEC-D-2020-1222). It was conducted in accordance with the tenets of the Declaration of Helsinki and conforms with the principles and applicable guidelines for the protection of human subjects in biomedical research.

The institutional medical records of pediatric (ages, 3–16 years) patients with aphakia, examined between January 2016 and December 2018, were retrospectively analyzed. The following parameters were included for the aphakic eyes: axial length [measured using an IOLMaster (Zeiss, Oberkochen, Germany)], keratometry value [measured using an automatic refractometer (KR-8900, Topcon, Tokyo, Japan)], and refractive values for distance [spherical equivalent, measured using retinoscopy and an automatic refractometer (KR-8900, Topcon)]. In all cases, retinoscopy was performed by experienced pediatric ophthalmologists and completed prior to automatic refractometer and axial length measurements. Refractometer measurements were repeated at least three times or until the standard deviation was <1 D. For axial length measurements, each eye was measured at least five times and each signal-to-noise ratio was >1.6. The mean values for the repeated measurements were reported.

The refractive statuses of each eye, obtained using three different methods (retinoscopy, automatic refractometer, vergence formula), were compared. The correlation coefficients for the established values were determined using regression analysis and the Bland-Altman model (Statistical Package for the Social Sciences, version 23; SPSS, Chicago, IL, USA). A *P*-value of <0.05 was considered statistically significant (12).

## Results

In total, 47 patients (72 eyes) were included in this study. There were 22 females and 25 males. The mean age of the patients at the time of examination (range) was 7 (2–16) years. The general descriptions of the patients and their eyes are summarized in Table 1. There were 39 eyes from patients aged between 2 and 6 years, 26 eyes from patients aged between 7 and 11 years and 7 eyes from patients aged between 12 and 16 years. Age had no significant effect on the measurement results.

**Table 1:** Baseline characteristics of the patients and eyes included in the study.

**Age at surgery**	**(Months)**
Mean ± SD,(Range)	21.75 ± 33.31, (2–114)
**Follow-up interval**	**(Months)**
Mean ± SD, (Range)	59.21 ± 35.27 (1–151)
Gender (female/male)	22/25
No. of eyes included	72
**Causes of aphakia**	**No. of eyes (% in the cohort)**
Congenital cataract	54 (75%)
Persistent hyperplastic primary vitreous	7 (9.3%)
Retinal detachment	4 (5.6%)
Familial exudative vitreoretinopathy	1 (1.4%)
Retinopathy of prematurity	1 (1.4%)
Retinoblastoma	1 (1.4%)
Congenital retinoschisis	2 (2.8%)
Lens dislocation(Hyperhomocysteiemia)	1 (1.4%)
Ocular toxocariasis	1 (1.4%)
**Major associated signs and symptoms**	**No. of eyes (% in the cohort)**
Nystagmus	38 (52.8%)
Microphthalmia	18 (25%)
Iris coloboma	10 (13.9%)
**Biometry measurement**	
Keratometry	(D as Average of K1 and K2)
Mean ± SD, (Range)	44.97 ± 2.72, (40.25–55.00)
**Axial length**	**(mm)**
Mean ± SD, (Range)	22.39 ± 2.07, (17.36–27.93)

*SD, standard deviation*.

**Figure 2:**
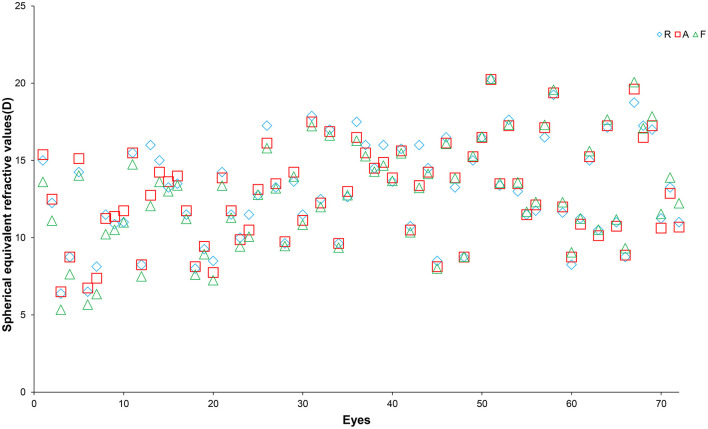
Spherical equivalent refractive values of the 72 eyes included in this study measured using a refractometer (R, blue diamonds), automatic refractometer (A, red squares), and the vergence formula (F, green triangles). The X-axis represents individual eyes and the Y-axis represents the refractive values (in dioptres).

**Figure 3:**
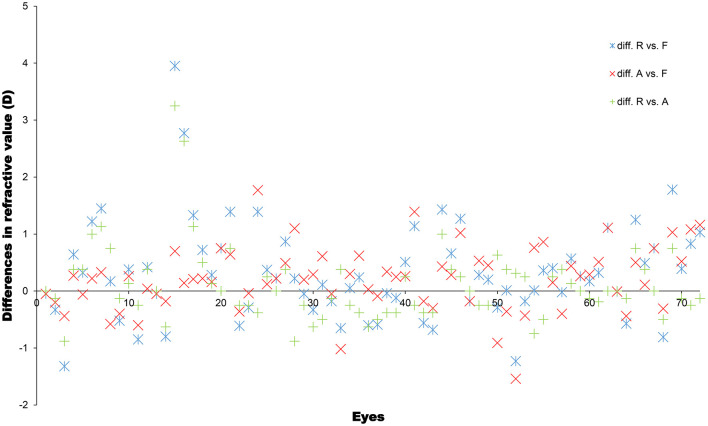
Differences in spherical equivalent refractive values (in dioptres), determined using a refractometer (R), automatic refractometer (A), and the vergence formula (F), for each eye.

Figure 2 shows the spherical equivalent refraction values obtained using the retinoscopy, automatic refractometer, and vergence formula methods for each eye. To better visualize the dioptre differences obtained using the different methods, the differences were plotted in Figure 3. Table 2 summarizes the overall average refractive values obtained using each method and the dioptre differences between different methods. It is observed that the results of the three methods are very close. Most of the differences are within 1D. In order to find out if corneal curvature or axial length may affect the differences between different methods, we divided eyes by corneal curvature: the curvier group with curvature of <45D and the flat group of more than 45D, and by the axial length: the short group of <22 mm, the average group of 22mm to 26mm, and the long group of more than 26. We then calculated the average differences between different methods in each group and found that neither corneal curvature nor axial length caused significant differences (data not shown). The results suggested that the vergence formula is reliable over a broad range of ocular biometry.

**Table 2:** Mean spherical equivalent refractive values (in diopters) obtained by retinoscopy (R), automatic refractometry (A) and vergence formula (F), and the differences between each other.

	***N***	**Mean**	**SD**	**Min**	**Max**	**95% CI**
R	72	13.01	3.27	6.38	20.25	12.28–13.72
A	72	12.90	3.23	6.50	20.25	12.18–13.63
F	72	12.70	3.40	5.34	20.30	11.92–13.45
diff. R vs. A	72	0.11	0.67	−0.88	3.25	−0.04–0.27
diff. R vs. F	72	0.31	0.86	−1.32	3.95	0.14–0.52
diff. A vs. F	72	0.21	0.56	−1.54	1.77	0.08–0.34
abs.diff. R vs. A	72	0.44	0.51	0.01	3.25	0.34–0.57
abs.diff. R vs. F	72	0.64	0.65	0.01	3.95	0.51–0.80
abs.diff. A vs. F	72	0.46	0.38	0.01	1.77	0.38–0.55

*diff., difference; abs.diff., the absolute difference; Min, minimal; Max, maximal; CI, confidence interval; N, number of eyes measured*.

**Figure 4:**
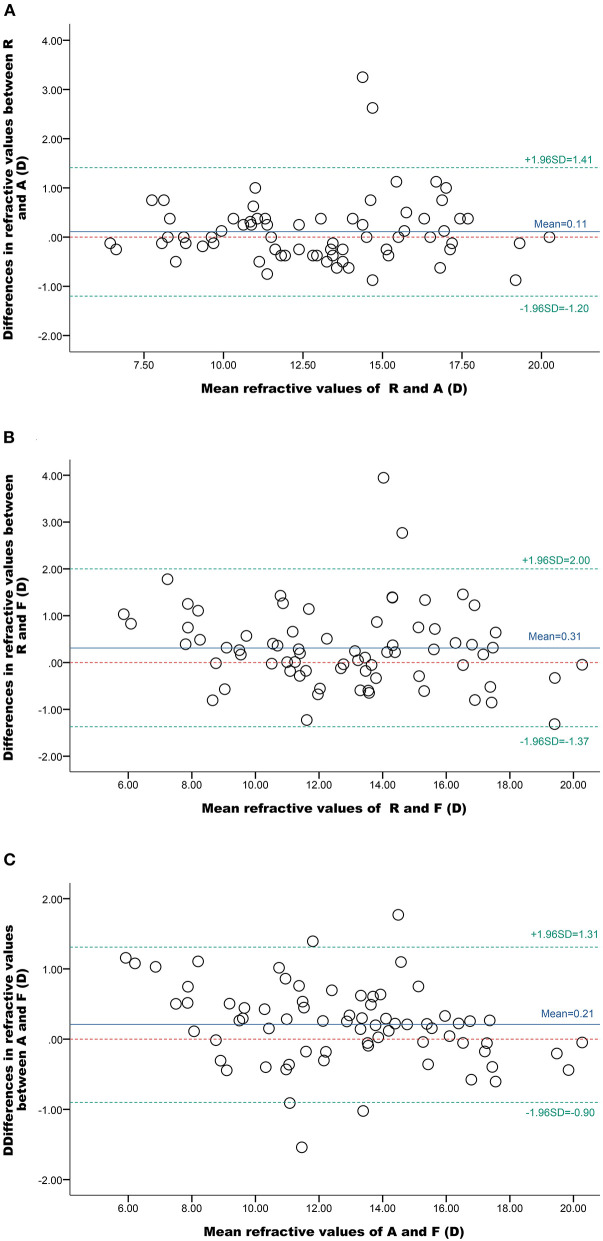
Bland-Altman plots of the spherical equivalent refractive values obtained using different methods. The solid blue lines represent the observed mean agreement between the methods, the dashed green lines represent the 95% limits of agreement, and the red, dashed, horizontal lines represent the perfect mean agreement between the methods. **(A)** Comparison of values determined using a refractometer (R) and an automatic refractor meter (A), **(B)** a refractometer (R) and the vergence formula (F), and **(C)** an automatic refractor meter (A) and the vergence formula (F).

To further assess the consistency of the refractive power values obtained using the three methods, a Pearson's correlation analysis was performed. The correlation coefficients were calculated for the results obtained using retinoscopy and the vergence formula [0.968; (y = 0.931x + 1.193), *P* < 0.001, *R*^2^ = 0.936], automatic refractometry and the vergence formula [0.987; (y = 0.937x + 1.004), *P* < 0.001, *R*^2^ = 0.974], and automatic refractometry and retinoscopy [0.979; (y = 0.992x + 0.215), *P* < 0.001, *R*^2^ = 0.958]. The Bland-Altman consistency analysis revealed that the mean differences in the spherical equivalent values between retinoscopy and automatic refractometry, retinoscopy and the vergence formula, and automatic refractometry and the vergence formula were 0.11 D, 0.31 D, and 0.21 D, respectively, with 95% limits of agreement of−1.20 to 1.41 D,−1.37 to 2.00 D, and−0.90 to 1.31 D, respectively (Figure 4, Table 3). Therefore, the three methods gave consistent results and appeared interchangeable.

**Table 3:** Bland-Altman consistency analysis.

	**R with A**	**R with F**	**A with F**
Avg. of diff.	0.11	0.31	0.21
95% LoA	−1.20–1.41	−1.37–2.00	−0.90–1.31
Data points beyond the consistency limit	2.78% (2/72)	2.78% (2/72)	6.94% (5/72)
Absolute value of the maximum in the consistency limit	1.13	1.78	1.16

*R, spherical equivalent refractive values obtained by retinoscopy; A, spherical equivalent refractive values obtained by automatic refractometer; F, refractive values calculated according to the vergence formula. diff., difference; LoA, limits of agreement*.

In addition to the 72 eyes described above, there were 9 eyes of 5 patients which had refractive values determined by either retinoscopy or automatic refractometry, but not both, due to the problems of the affected eyes. We also calculated refractive values using vergence formula. The results were shown in Table 4. With the exception of 1 eye, the rest showed good agreement between measurements with differences <1D. The result again demonstrated that the vergence formula was a valuable method for estimating the refractive status of aphakic eyes.

**Table 4:** Comparison of refractive values calculated by vergence formula with values obtained by either retinoscopy or automatic refractometer in eyes where the measurement for both was not possible.

**Patient**		**Automatic**		**Axial length**	**Axial length (by**		**Diff. RvsF**
**no**.	**Retinoscopy (R)**	**refractometer (A)**	**Keratometry**	**(by IOLmaster)**	**type A ultrasonic)**	**Formula (F)**	**or AvsF**
A	The pupil was too small	16.13	46.63	20.03	None	16.18	−0.05
B	The pupil was too small	16.00	46.13	20.41	None	15.69	0.31
C	19.5	Poor fixation	46.63	Poor fixation	18.40	19.81	−0.31
D	19.5	Poor fixation	47.13	Poor fixation	18.80	18.60	0.90
E	10.25	Pupil irregularity	44.77	23.87	None	9.88	0.37
F	10	Poor fixation	46.25	Poor fixation	23.60	9.21	0.79
G	10	Poor fixation	46.13	Poor fixation	23.70	9.12	0.88
H	24.25	Beyond measuring range	46.13	16.62	None	24.28	−0.03
I	26	Beyond measuring range	46.13	16.80	None	23.84	2.16

## Discussion

In children with aphakia, accurate measurements of the refractive errors of their eyes is difficult, and multiple approaches are preferred to achieve better refractive status assessments. This study proposed a formula, based on the spherical features of the cornea and the axial length, to objectively calculate the refractive status of pediatric aphakic eyes. The refractive error calculated using this formula agreed with the values determined using the traditional methods of retinoscopy and automatic refractometry; therefore, this may be a useful supplementary method in optometry.

The main advantages of this method are its objectivity and ease of use. These features are desirable when measuring eyes with high refractive errors, poor coordination, or irregular pupils and corneas (13). The formula requires the input of the axial length, which can be obtained using a variety of methods. For example, axial length can be measured using an IOLMaster or A-scan, B-scan, or immersion ultrasonic measurement devices, with or without anesthesia. Axial length determinations using an IOLMaster or immersion ultrasound are, reportedly, almost equivalent (14). The IOLMaster tends to yield measurements that are 0.15 mm longer than those determined using A-scan ultrasonography (15).

The vergence formula is particularly valuable when the conventional method is inadequate for analyzing the refractive power of an eye. As seen in the patients included in Table 4, the vergence formula complimented the retinoscopy limitation observed in a patient with a very small pupil and in several patients with irregular pupils, poor fixation, and refractive errors greater than the measurement range of the automatic refractometer. In patients with irregular pupils, retinopathy, nystagmus, and visual axis deviation, the results of retinal imaging can be greatly disturbed (16).

The vergence formula utilizes the relationships among corneal curvature, axial length, and refractive status of aphakic eyes. Only two of them are needed to estimate the last one. Khan (17) also reported a method to calculate axial lengths using spherical equivalent values. These parameters are very important for the rehabilitation of children with aphakia who undergo intraocular lens implantation.

The formula, $F_{C}=\frac{n_{a}}{L}-K$, can also be used to prescribe corneal contact lenses; the distance conversion between the lens and cornea can be ignored. For young children with aphakic eyes, a +32 D silicone hydrogel soft lens (175 USD) is often a suggested treatment until a more accurate refractive error value can be measured. Thus, the formula used in the present study may provide a more accurate assessment of the refractive errors in young children, reducing the frequency of contact lens changes and increasing patient compliance (18, 19).

It should be noted that changes in the contents of the vitreous would lead to the corresponding changes in the index of reflection. For example, a silicone oil-filled eye after retinal surgery would have different index of reflection than aqueous media. However, silicone oil is emulsified and needs to be removed after a period of time. Theoretically, the formula should still work on silicone oil-filled eye if we change to the index of reflection to that of the silicone oil. However, this needs to be tested in future studies.

The limitations of this study included the relatively small number of patients and the preponderance of children at approximately 2 years of age. Future studies need to include patients representing a greater diversity of ages, including new-borns and adults. Such studies are necessary to further validate the accuracy of the formula. Furthermore, the present formula uses typical values for the refractive indexes of the cornea and vitreous, ignoring individual differences.

## Conclusion

In summary, this study suggests that a vergence formula calculation may be an alternative for assessing the refractive status of the aphakic eye in pediatric patients.

## Data Availability Statement

The original contributions presented in the study are included in the article, further inquiries can be directed to the corresponding author.

## Ethics Statement

The studies involving human participants were reviewed and approved by the Ethics Committee of Xinhua Hospital, Affiliated with the Shanghai Jiao Tong University School of Medicine. Written informed consent to participate in this study was provided by the participants' legal guardian/next of kin.

## Author Contributions

LT: conceptualization, data curation, formal analysis, and investigation. PZ, HZ, XK, and YW: data curation. LC: data curation and formal analysis. JL: funding acquisition, supervision, and writing–original draft. All authors contributed to the article and approved the submitted version.

## Funding

This research was supported by grants from the Ministry of Science and Technology of China (2018YFA0800801) and from the National Natural Science Foundation of China (81873679).

## Conflict of Interest

The authors declare that the research was conducted in the absence of any commercial or financial relationships that could be construed as a potential conflict of interest.

## Publisher's Note

All claims expressed in this article are solely those of the authors and do not necessarily represent those of their affiliated organizations, or those of the publisher, the editors and the reviewers. Any product that may be evaluated in this article, or claim that may be made by its manufacturer, is not guaranteed or endorsed by the publisher.
